# Short-Term Effects of Spinal Manual Therapy on the Nervous System in Managing Musculoskeletal Pain: A Systematic Review

**DOI:** 10.3390/jcm14113830

**Published:** 2025-05-29

**Authors:** Chloé Jupin, Vicente Beltran Aibar, François-Régis Sarhan

**Affiliations:** 1 Physiotherapy School, Centre Hospitalier Universitaire Amiens-Picardie, 80054 Amiens, France; beltranaibar.vicente@chu-amiens.fr (V.B.A.); sarhan.francois-regis@chu-amiens.fr (F.-R.S.); 2 Institut D’ingénierie de la Santé, Université de Picardie Jules Verne, 80000 Amiens, France; 3 UR 7516 CHIMERE, Université Picardie Jules Verne, 80000 Amiens, France

**Keywords:** nervous system, central nervous system, autonomic nervous system, manual therapy, musculoskeletal pain

## Abstract

**Background**: Spinal manual therapy (SMT) is widely used in the management of musculoskeletal pain. In addition to mechanical effects, SMT may induce neurophysiological changes at both central and autonomic levels. However, the extent and consistency of these short-term effects remain unclear. **Objective**: To systematically review the short-term effects of SMT on pain perception, central nervous system (CNS) activity, and autonomic nervous system (ANS) responses in adults with musculoskeletal pain or in healthy controls. **Methods**: A systematic review was conducted. Three databases (PubMed, ScienceDirect, Embase) were searched up to October 2023, with a final update in March 2025. Randomized controlled trials involving SMT and assessing outcomes related to pain, CNS, or ANS function were included. The methodological quality was assessed using the PEDro scale. The results were synthesized narratively and categorized by outcome domain. Four summary tables were created to present the study characteristics, main findings, methodological quality, and risk of bias. **Results**: Eleven trials were included. SMT produced variable effects on pain perception, with more consistent results observed when the treatment was applied frequently and followed standardized protocols. The CNS-related outcomes (e.g., fMRI connectivity, motor-evoked potentials) suggested short-term modulation of brain and spinal excitability in some studies. The ANS responses were heterogeneous, ranging from parasympathetic activation to sympathetic stimulation, depending on the intervention and population. The methodological quality was moderate to high in most studies, although the small sample sizes and limited blinding increased the risk of bias. The effect sizes were not consistently reported. **Conclusions**: SMT may induce short-term neuromodulatory effects on pain, CNS, and ANS activity. These effects appear to be context-dependent and require precise, repeated, and purposeful application.

## 1. Introduction

Manual therapy (MT) is a specialized domain within physiotherapy dedicated to treating neuromusculoskeletal (NMS) disorders through techniques such as mobilizations and manipulations. MT practice is grounded in both manual techniques and clinical reasoning. The commonly employed methods include joint mobilizations, high-velocity low-amplitude (HVLA) thrust manipulation, mobilizations with movement (Mulligan), repeated movement-based exercises (McKenzie), neurodynamic techniques, endurance-based approaches strategies, and trigger point therapy [1]. These techniques are embedded within conceptual frameworks that guide assessment and decision-making processes. Six major models shape current MT practice: the Cyriax approach, the McKenzie method, the Kaltenborn approach, the Mulligan concept, the Maitland concept, and neurodynamic theory [2].

In this context, we specifically focus on spinal MT, which encompasses mobilizations and manipulations applied to the vertebral column. The Maitland and Mulligan approaches hold particular significance. The Maitland approach utilizes a five-grade system of mobilizations, ranging from grade I to grade IV, with grade V corresponding to manipulation. Grades I and II are applied within the pain-free range of motion, while grades III, IV, and V are employed at the end range of motion with resistance [3]. In contrast, the Mulligan concept aims to restore pain-free movement by applying a sustained glide perpendicular to the plane of physiological motion during active movement.

**Figure 1:**
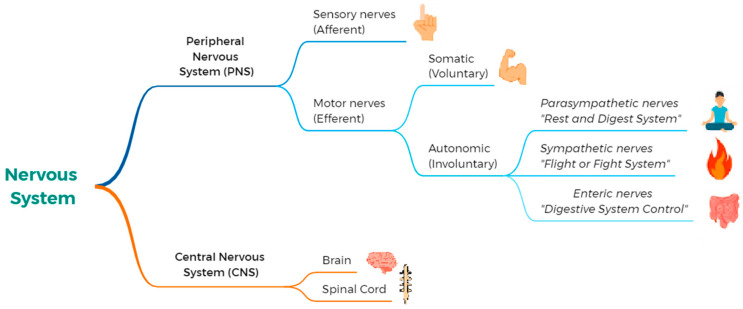
Hierarchical organization and functional subdivisions of the human nervous system.

The nervous system is a complex structure comprising the central nervous system (CNS) and the peripheral nervous system (PNS). The CNS includes the brain and spinal cord, which are responsible for information integration and the regulation of bodily functions. The PNS consists of cranial and spinal nerves that relay information to and from the CNS. It is divided into two branches: the somatic nervous system, which governs the voluntary control of skeletal muscles; and the autonomic nervous system (ANS), which operates involuntarily to regulate functions such as heart rate and digestion. To support the interpretation of the outcomes related to CNS and ANS function, a summary diagram of the human nervous system is presented (Figure 1).

The ANS includes the sympathetic and parasympathetic systems, which have opposing actions, as well as the enteric nervous system, which controls the digestive system [4]. The CNS also plays a key role in chronic pain through the involvement of the limbic system, which modulates pain perception [5]. Structural and functional alterations in brain areas such as the prefrontal cortex, anterior cingulate cortex, and periaqueductal gray may contribute to the persistence of pain [6,7]. In patients with chronic pain, the brain activation patterns differ significantly, often favoring emotional and affective processing over sensory processing [8]. These phenomena, associated with central sensitization, may lead to long-lasting changes in CNS structure and function, ultimately altering pain modulation processes [9,10].

Although MT is widely recognized for its effectiveness in musculoskeletal pain management, the mechanisms underlying its effects remain poorly understood [9]. Most studies have focused on symptom relief without thoroughly exploring how spinal MT (SMT) influences CNS or ANS activity. Moreover, the substantial variability in the techniques, treatment protocols, and outcome measures across studies limits the comparability and generalization of the findings [10].

This systematic review aims to evaluate the short-term effects of SMT on pain perception and nervous system activity, with particular attention given to both central and autonomic components. By synthesizing the current evidence on neurophysiological outcomes, we seek to identify patterns that may inform clinical practice and guide future research.

## 2. Materials and Methods

### 2.1. Study Design and Registration

This systematic review was conducted in accordance with the Preferred Reporting Items for Systematic Reviews and Meta-Analyses (PRISMA 2020) guidelines [11], and was prospectively registered on PROSPERO (registration number CRD42023464257).

### 2.2. Search Strategy

A comprehensive literature search was performed in three electronic databases—PubMed, ScienceDirect, and Embase—to identify relevant studies evaluating the short-term effects of spinal manual therapy on pain and nervous system function. The initial search included all studies published up to October 2023, and a final update was conducted in March 2025 to ensure the inclusion of recent data. The search strategy combined free-text terms and controlled vocabulary (e.g., MeSH terms), adapted to each database. For example, the PubMed query was *(“spinal manual therapy” OR “spinal manipulation” OR “mobilization”) AND (“nervous system” OR “pain” OR “autonomic nervous system” OR “central nervous system”).* Complete search strategies for all databases are provided in the Table A1.

### 2.3. Eligibility Criteria

The eligible studies were randomized controlled trials involving adult human participants (≥18 years) with musculoskeletal pain. The interventions had to include at least one spinal manual therapy technique—mobilization or manipulation—and assess outcomes related to pain perception or nervous system activity (central or autonomic). Only studies published in English or French were included. The exclusion criteria were studies involving pediatric populations, participants with chronic systemic or neurological disorders, studies using non-spinal or non-manual interventions, or those not reporting pain or neurophysiological outcomes.

### 2.4. Selection Process

**Figure 2:**
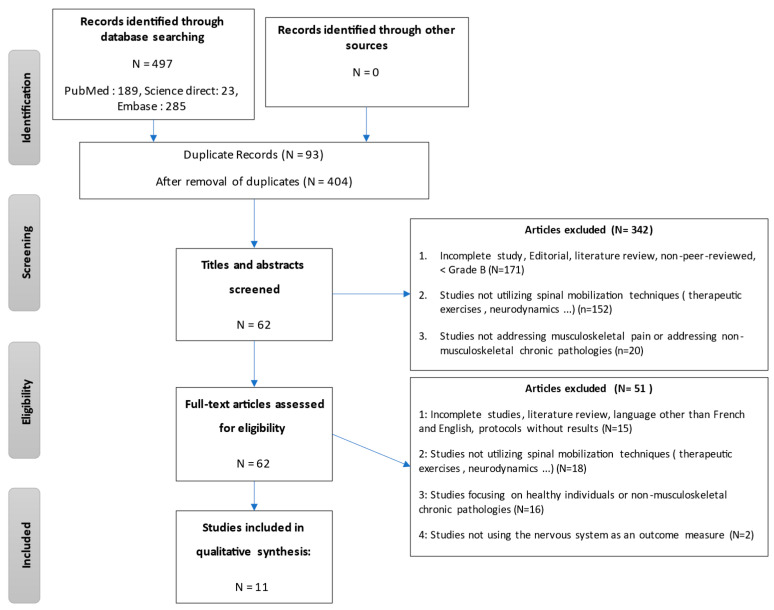
PRISMA flowchart of the study selection process.

Two reviewers independently screened the titles and abstracts, followed by a full-text review of potentially eligible studies. Disagreements were resolved through discussion or consultation with a third reviewer. The selection process followed the PRISMA guidelines, and is illustrated in the PRISMA flow diagram (Figure 2).

### 2.5. Data Extraction

The data were independently extracted by two authors using a standardized form. The extracted variables included the authorship, year of publication, sample size, population characteristics (e.g., age, sex, anatomical site of pain), details of the manual therapy protocol (technique, frequency, duration), comparator intervention (if applicable), outcome measures (e.g., pain scales, physiological variables, neuroimaging markers), and key findings. When available, statistical data such as the means, standard deviations, *p*-values, and effect sizes were recorded. Discrepancies were resolved by discussion.

### 2.6. Methodological Quality Assessment and Risk of Bias

The methodological quality of each included study was assessed using the PEDro scale, which comprises 11 items evaluating internal validity and statistical reporting. As the first item (related to eligibility criteria) is not included in the final score, the maximum score is 10 points. Studies with a score ≥ 6 were considered to be of moderate to high quality. The PEDro scores are presented in Table A2 and were also used to estimate the level of evidence. Methodological limitations, including small sample sizes, lack of blinding, and variability in intervention protocols, were discussed qualitatively.

### 2.7. Data Synthesis

Due to heterogeneity in the interventions and outcome measures, no meta-analysis was conducted. Instead, a narrative synthesis was performed. The results were grouped by outcome domain into pain perception, central nervous system activity, and autonomic nervous system function. Consequently, potential publication bias was not formally assessed using funnel plots or statistical methods.

## 3. Results

### 3.1. Study Selection

A total of 404 articles were initially identified through database searching. After the removal of duplicates and screening of titles and abstracts, 43 full-text articles were assessed for eligibility. Ultimately, 11 randomized, controlled trials met the inclusion criteria and were included in this review. The study selection process is detailed in the PRISMA flowchart (Figure 2) and PRISMA checklist (Figure S1).

### 3.2. Study Characteristics

The main characteristics of the included studies are presented in Table 1. The study populations included adults with musculoskeletal pain in various regions (neck, lower back, ankle, shoulder, craniofacial) and healthy volunteers. The sample sizes ranged from 20 to 80 participants. The interventions consisted of spinal manual therapy techniques, including mobilizations (e.g., Maitland, Mulligan) and high-velocity low-amplitude (HVLA) manipulations, sometimes combined with other modalities such as therapeutic messaging or stretching exercises. The treatment durations varied from a single session to repeated applications over 2 to 8 weeks. The primary outcomes measured were related to pain perception, central nervous system (CNS) activity, and autonomic nervous system (ANS) function, using tools such as numerical rating scales, pressure pain thresholds (PPT), heart rate variability (HRV), skin conductance, functional MRI, and motor-evoked potentials (MEPs).

### 3.3. Main Findings

An overview of the key outcomes and their statistical significance is provided in Table 2, along with the domains assessed and effect size reporting.

Out of the seven studies that assessed pain using the NRS, three reported a significant decrease in pain following manual therapy (*p* < 0.05), notably the studies by Haider et al. [13], La Touche et al. [14], and Weber et al. [18]. Other studies reported a reduction in pain across all intervention groups, without statistically significant between-group differences. However, Bialosky et al. [12] observed a greater decrease in pain intensity in the group that received contextual verbal suggestions, referred to as “positive therapeutic messaging”, delivered alongside manual therapy. This highlights the potential role of expectancy effects in modulating clinical outcomes.

Regarding the pressure pain threshold (PPT), only La Touche et al. [14] found a significant increase following manual therapy. Other studies (Bialosky, Gay, Peña-Salinas [10,12,14]) reported either no change or non-significant results. The effectiveness of manual therapy on thermal pain thresholds was only assessed in one study (Bialosky [12]), which showed a reduction in pain but no intergroup difference.

In terms of central nervous system activity, four studies reported measurable effects. Gay et al. [10] observed increased functional connectivity in the HVLA group between the somatosensory cortex and pain-related regions. Weber et al. [18] demonstrated reduced activation in neurologic pain signature areas (e.g., ACC). Fisher et al. [9] found increased MEPs in the tibialis anterior after manual therapy, suggesting a temporary window of increased excitability. These changes may support neurophysiological modulation beyond analgesia.

Concerning the autonomic nervous system, five studies assessed HRV or related indicators. While Rodrigues et al. [16] found greater parasympathetic activation in the spinal manipulation group (*p* < 0.05), Bakken et al. [6] observed no significant changes when combining manipulation with exercise. Conflicting results were also seen in heart rate and respiratory rate results, with La Touche [14] reporting sympathetic activation and Barassi indicating parasympathetic dominance. Sillevis et al. [17] explored pupil diameter variations in relation to audible joint sounds and found trends suggesting different ANS responses depending on acoustic cues.

### 3.4. Methodological Quality

The PEDro scores for the 11 studies ranged from 5 to 8 out of 10, as shown in Table 3. Eight studies were rated as having moderate to high quality (PEDro ≥ 6), with good internal validity and appropriate reporting of outcomes. However, blinding was frequently lacking, especially among therapists and participants, which increases the risk of performance bias.

### 3.5. Risk of Bias

A qualitative assessment of potential sources of bias is presented in Table 4. The common limitations included small sample sizes (n ≤ 30 in 7 studies), a lack of allocation concealment, and the absence of participant or therapist blinding. Despite these limitations, three studies were assessed as having low overall risk of bias, while most others were considered to have a moderate risk, and two were deemed to present a high risk of bias due to methodological weaknesses.

## 4. Discussion

### 4.1. Interpretation of Findings

Pain perception was the most frequently studied outcome. Notably, the studies with the largest sample sizes and highest methodological quality (e.g., Bialosky et al., n = 80; Bakken et al., n = 50) did not report significant between-group differences. This pattern suggests that the effects of SMT may be sensitive to contextual factors and the study design, and that positive findings in smaller trials should be interpreted with caution, given the increased risk of type I error. These positive results were particularly pronounced in studies involving frequent sessions and structured protocols, such as La Touche et al. [14], who demonstrated significant improvements in pressure pain threshold and pain intensity following a highly standardized cervical mobilization protocol. In contrast, studies such as that by Bialosky et al. [12], although reporting improvements in all groups, found no difference between SMT and placebo, unless the intervention was accompanied by “positive therapeutic messaging”, underscoring the importance of contextual and communicational factors in pain modulation. These findings are consistent with previous evidence suggesting that placebo responses and therapeutic expectations can significantly influence outcomes in manual therapy, as shown by Jensen et al. [19].

The effects on the central nervous system (CNS) were supported by several studies showing neurophysiological changes following SMT. Gay et al. [10] reported increased functional connectivity in the somatosensory and insular regions after HVLA thrusts, while Weber et al. [18] observed a reduction in activity within neurologic pain signature regions, particularly the anterior cingulate cortex. These results align with imaging studies showing that chronic pain alters the activity of limbic and prefrontal brain areas involved in the affective processing of pain [5,8]. Furthermore, Fisher et al. [9] reported increased motor-evoked potentials post-intervention, suggesting a temporary enhancement of corticomotor excitability that may facilitate motor control rehabilitation.

In contrast, the autonomic nervous system (ANS) responses to SMT were more variable. Some studies, such as that by Rodrigues et al. [16], found evidence of parasympathetic activation through increased HRV and decreased systolic blood pressure values. Conversely, La Touche et al. [14] reported sympathetic activation following cervical mobilizations, reflected in increased heart rate, respiratory rate, and skin conductance values. These divergent results may reflect differences in the duration, intensity, or frequency of SMT, or in the underlying physiological states of the participants. Barassi et al. [7], for instance, used eight weekly sessions and found significant reductions in heart rate and respiratory rate, interpreted as markers of parasympathetic dominance. These findings echo prior evidence from massage therapy research suggesting that low-intensity tactile stimulation may enhance high-frequency HRV components and promote relaxation [20].

The variability observed in the autonomic responses also points to broader methodological issues, such as the variety of measurement tools and the lack of standardization between studies. Although several studies reported effect sizes, these values were highly variable and occasionally unexpectedly large, limiting their interpretability. In many cases, the context in which the effect size was measured, such as a small sample size or single-session intervention, undermines its clinical relevance. While the inclusion of these values adds useful information, the overall picture remains too inconsistent to draw firm conclusions about the true impact of spinal manual therapy on autonomic function.

The clinical guidelines for the management of lower back pain, such as those published by George et al. [21], support the short-term use of manual therapy techniques for pain reduction. These recommendations, based on high-quality evidence are consistent with our findings that SMT can produce immediate analgesic effects but also stress the need for combination with active approaches for long-term impact.

### 4.2. Relevance to Clinical Practice

Spinal manual therapy appears to offer immediate pain relief and may influence neurophysiological processes, particularly when applied with intention and precision. However, the evidence reviewed here indicates that SMT is not sufficient as a standalone intervention. Its effects are most pronounced when it is part of a structured, repeated, and individualized treatment plan, rather than used in isolation or routine practice.

For clinicians, SMT should be viewed as a tool that creates short-term neurophysiological windows of opportunity, which can be strategically used to facilitate movement, reinforce therapeutic education, or prepare the patient for active treatment. The clinical context—including patient expectations and therapist communication—also appears to play a critical role in shaping outcomes. Pain relief should, therefore, be considered not solely as a biomechanical phenomenon but as the result of targeted stimulation within a meaningful therapeutic interaction.

In practical terms, the key message is: “Manual therapy works best when it is precise, repeated, and purpose-driven”. When used in this way, SMT can enhance patient engagement and optimize the short-term outcomes, particularly when combined with education and movement-based strategies.

### 4.3. Recommendations for Future Research

Future research studies should strive for methodological consistency across trials. The protocols should clearly define the type, frequency, and duration of SMT used, and the sample sizes should be sufficient to ensure statistical power. In addition, neurophysiological tools such as fMRI, HRV, or electrodermal activity should be integrated more widely to objectively assess central and autonomic changes. It would also be valuable to explore how different patient phenotypes—such as those with high pain sensitivity, catastrophizing tendencies, or specific postural patterns—respond to SMT. Lastly, greater methodological rigor, including concealed allocation and adequate blinding, remain essential to reduce bias and improve the internal validity.

### 4.4. Limitations

Several limitations affect the present review. Many included studies had small sample sizes, reducing the precision of the estimated effects. Blinding was rarely applied to therapists or participants, introducing potential performance and detection bias. The diversity in techniques, populations, and outcome measures precluded a meta-analysis and hindered direct comparisons.

## 5. Conclusions

This systematic review suggests that spinal manual therapy may induce short-term changes in pain perception and neurophysiological activity. However, the findings remain inconsistent and limited by the methodological heterogeneity and small sample sizes. SMT may serve as a transient modulatory stimulus when used within a multimodal and clinically reasoned approach.

## Figures and Tables

**Table 1:** Characteristics of included studies evaluating the short-term effects of spinal manual therapy.

Study	Population/Sample	Intervention	Frequency/Duration	Control/Comparator	Outcome Measure
Bakken et al. (2021) [6]	Patients with neck pain/n = 50	Spinal manipulation + stretching	3×/week for 2 weeks	Stretching only	Heart rate variability (HRV)
Barassi et al. (2018) [7]	Healthy adults/n = 20	Manual therapy on lumbar spine	1×/week for 8 weeks	Massage	Pain (NRS), postural control, HR, RR, SpO_2_, CO_2_
Bialosky et al. (2014) [12]	Lower back pain/n = 80	Spinal manipulation + messages	3×/week for 2 weeks	Placebo + message/No treatment	Pain sensitivity (PPT), thermal threshold, satisfaction
Fisher et al. (2016) [9]	Ankle sprain history/n = 24	Joint mobilization (varied velocities)	Single session	None	Motor-evoked potentials (MEPs)
Gay et al. (2014) [10]	Induced lower back pain/n = 24	Manual therapy session	Single session	Sham MT	fMRI connectivity, pain (NRS), PPT
Haider et al. (2018) [13]	Subacromial pain/n = 22	Maitland thoracic manipulation	3×/week for 2 weeks	Conservative exercise therapy	Pain (NRS), Shoulder Function Score
La Touche et al. (2013) [14]	Cervico-craniofacial pain/n = 22	Upper cervical mobilization	3×/week for 2 weeks	Placebo mobilization	PPT, pain (NRS), skin conductance, HR, RR, temperature
Peña-Salinas et al. (2017) [15]	Cervical whiplash/n = 30	First rib manipulation	Single session	None	PPT (nerve and muscle)
Rodrigues et al. (2021) [16]	Musculoskeletal pain/n = 30	Spinal manipulation	Single session	Sham MT	HRV, blood pressure, HR
Sillevis et al. (2011) [17]	Neck pain/n = 20	Thoracic manipulation	Single session	Sham manipulation	Pupil diameter, pain (NRS)
Weber et al. (2019) [18]	Neck pain/n = 24	Thoracic manipulation	Single session	Sham manipulation	Neurological activation zones, Pain (NRS)

NRS: Numerical Rating Scale; PPT: Pressure Pain Threshold; HR: Heart Rate; RR: Respiratory Rate; HRV: Heart Rate Variability; SpO_2_: Oxygen Saturation; CO_2_: Carbon Dioxide; fMRI: Functional Magnetic Resonance Imaging; MEPs: Motor-Evoked Potentials; MT: Manual Therapy.

**Table 2:** Summary of main findings from included studies assessing the short-term effects of spinal manual therapy (SMT) on pain perception and nervous system function.

Study	Key Outcomes	Statistical Significance	Domains Assessed	Effect Size
Bakken et al. (2021) [6]	No significant difference in HRV	Not significant	SNA (HRV)	Not reported
Barassi et al. (2018) [7]	Significant ↓ in pain and RR; improved postural control	*p* < 0.05	Pain, SNA (HR, RR), postural control	Pain: d = 2.75; RR: d = 3.00; Postural control: d = 0.47
Bialosky et al. (2014) [12]	↓ in pain (all groups); greater effect with contextual verbal suggestion (“positive message”)	*p* < 0.05 (positive message condition)	Pain, Pain sensitivity (PPT), Placebo effect	η^2^ = 0.17 (message effect on pain)
Fisher et al. (2016) [9]	↑ in passive & active MEPs (tibialis anterior)	*p* < 0.05	CNS (MEPs)	d = 0.90 (MEPs amplitude)
Gay et al. (2014) [10]	↑ in functional connectivity (HVLA group); ↓ pain	*p* < 0.05 (group × time interaction)	Pain, CNS (fMRI connectivity)	η = 0.24 (connectivity change)
Haider et al. (2018) [13]	↓ in pain and ↑ shoulder function (Maitland group)	*p* < 0.05	Pain, Function	Pain: d = 0.34–1.29; function: d = 0.34–1.66
La Touche et al. (2013) [14]	↑ PPT, ↓ pain, ↑ HR and RR, ↑ skin conductance	*p* < 0.05	Pain, PPT, ANS (HR, RR, skin conductance), CNS	Pain: d = 4.26; RR: d = 3.79; PPT: d = 3.34; HR: d = 2.68; skin conductance: d = 3.78
Peña-Salinas et al. (2017) [15]	No significant difference in PPT	Not significant	Pain sensitivity (PPT)	Not reported
Rodrigues et al. (2021) [16]	↓ sympathetic activity; ↑ parasympathetic (HRV); ↓ SBP	*p* < 0.05	ANS (HRV, HR, BP)	d = 0.40 (HRV, BP)
Sillevis et al. (2011) [17]	Pupil response differed by noise; ↓ pain observed	Mixed, trend only	Pain, ANS (pupil diameter)	d = 0.50 (pupil response to noise)
Weber et al. (2019) [18]	↓ in pain and in neurological activation (ACC)	*p* < 0.05	Pain, CNS (neurological activation)	d = 0.32 (pain and CNS activation)

CNS: Central Nervous System; ANS: Autonomic Nervous System; HRV: Heart Rate Variability; RR: Respiratory Rate; HR: Heart Rate; BP: Blood Pressure; PPT: Pressure Pain Threshold; MEPs: Motor-Evoked Potentials; fMRI: Functional Magnetic Resonance Imaging; ACC: Anterior Cingulate Cortex. Symbols: ↑ = increase; ↓ = decrease (compared to baseline or control condition).

**Table 3:** Methodological quality assessment using the PEDro Scale.

Study	PEDro Score (/10)	Quality Level	Blinding (Participants/Therapists/Assessors)
Bakken et al. (2021) [6]	8	High	No/No/Yes
Barassi et al. (2018) [7]	6	Moderate	No/No/Yes
Bialosky et al. (2014) [12]	8	High	Yes/No/Yes
Fisher et al. (2016) [9]	7	High	No/No/Yes
Gay et al. (2014) [10]	8	High	No/Yes/Yes
Haider et al. (2018) [13]	5	Low	No/No/Yes
La Touche et al. (2013) [14]	8	High	Yes/No/Yes
Peña-Salinas et al. (2017) [15]	8	High	No/No/Yes
Rodrigues et al. (2021) [16]	7	High	No/No/Yes
Sillevis et al. (2011) [17]	5	Low	Yes/No/No
Weber et al. (2019) [18]	8	High	Yes/Yes/No

**Table 4:** A risk of bias analysis based on methodological limitations.

Study	Sample Size (<30)	Randomization Reported	Allocation Concealed	Blinding Limitations	Overall Risk of Bias
Bakken et al. (2021) [6]	No	Yes	Yes	No blinding of participants or therapists	Low
Barassi et al. (2018) [7]	Yes	Yes	No	No blinding of participants or therapists	Moderate
Bialosky et al. (2014) [12]	No	Yes	Yes	No therapist blinding	Low
Fisher et al. (2016) [9]	Yes	Yes	No	No blinding of participants or therapists	Moderate
Gay et al. (2014) [10]	Yes	Yes	Yes	No participant blinding	Moderate
Haider et al. (2018) [13]	Yes	Yes	No	No blinding of participants or therapists	High
La Touche et al. (2013) [14]	Yes	Yes	Yes	No therapist blinding	Low
Peña-Salinas et al. (2017) [15]	No	Yes	Yes	No blinding of participants or therapists	Low
Rodrigues et al. (2021) [16]	No	Yes	Yes	No blinding of participants or therapists	Moderate
Sillevis et al. (2011) [17]	Yes	Yes	No	Only participant blinding	High
Weber et al. (2019) [18]	Yes	Yes	No	No assessor blinding	Moderate

## Data Availability

This article declares that the data are available. This systematic review, conducted in accordance with PRISMA guidelines, was registered on PROSPERO (Registration No. CRD42023464257).

## Supplementary Materials

The following supporting information can be downloaded at: https://www.mdpi.com/article/10.3390/jcm14113830/s1, Figure S1: PRISMA checklist [11].

## Appendix A

**Table A1:** Research formulation in PICO format and associated search equation (MeSH terms).

PICO	Formulation	Associated Keywords
Population	Adults experiencing musculoskeletal pain	Musculoskeletal pain
Intervention	Spinal manual therapy, including mobilization or manipulation	Manual therapy; spinal manipulation; musculoskeletal manipulation
Comparison	Comparison across included studies	–
Outcomes	Effects of spinal manual therapy on the nervous system and pain perception	Nervous system; autonomic nervous system; central nervous system; spinal cord; peripheral nervous system; neurophysiology; pain
((manual therapy) OR (musculoskeletal manipulation)) AND (musculoskeletal pain) AND (pain) AND ((nervous system) OR (autonomic nervous system) OR (central nervous system) OR (spinal cord) OR (peripheral nervous system) OR (neurophysiology))		

**Table A2:** PEDro scale scores for the 11 studies included in the systematic review.

Studies (Years)	1.	2.	3.	4	5.	6.	7.	8.	9.	10.	11.	Score Total (/10)
Bakken et al. (2021) [6]	*****	*****	*****	*****	**-**	**-**	*****	*****	*****	*****	*****	**8**
Barassi et al. (2018) [7]	**-**	*****	*****	*****	**-**	**-**	**-**	*****	*****	*****	**-**	**6**
Bialosky et al. (2014) [12]	*****	*****	*****	*****	*****	**-**	**-**	*****	*****	*****	*****	**8**
Fisher et al. (2016) [9]	**-**	*****	**-**	*****	**-**	**-**	*****	*****	*****	*****	*****	**7**
Gay et al. (2014) [10]	*****	*****	*****	*****	**-**	*****	*****	*****	*****	*****	**-**	**8**
Haider et al. (2018) [13]	**-**	*****	**-**	*****	**-**	**-**	**-**	*****	**-**	*****	*****	**5**
La Touche et al. (2013) [14]	*****	*****	*****	*****	*****	**-**	*****	*****	**-**	*****	*****	**8**
Peña-Salinas et al. (2017) [15]	*****	*****	*****	*****	**-**	**-**	*****	*****	*****	*****	*****	**8**
Rodrigues et al. (2021) [16]	*****	*****	*****	*****	**-**	**-**	*****	*****	**-**	*****	**-**	**7**
Sillevis et al. (2011) [17]	*****	*****	*****	*****	**-**	**-**	**-**	*****	**-**	*****	**-**	**5**
Weber et al. (2019) [18]	*****	*****	*****	*****	*****	*****	**-**	**-**	**-**	*****	*****	**8**

Symbol: “*” indicates that the criterion was fulfilled; “-” indicates that the criterion was not fulfilled. Criterion 1: Eligibility criteria specified (this criterion is not used to calculate the PEDro score). Criterion 2: Random allocation of participants. Criterion 3: Allocation concealed. Criterion 4: Groups similar at baseline regarding the most important prognostic indicators. Criterion 5: Participants blinded. Criterion 6: Therapists blinded. Criterion 7: Assessors blinded. Criterion 8: Measures of key outcomes obtained from more than 85% of subjects. Criterion 9: Data analyzed by intention to treat. Criterion 10: Between-group statistical comparisons were conducted. Criterion 11: Point measures and measures of variability were provided.

**Table A3:** Characteristics of the 11 studies included in the review using the FITT-VP model.

Studies	Pedro Scale	Duration	Intervention	Frequency (Intervention Duration)	Intensity	Groups Types	**Volume**	**Others**
Bakken et al. [6]	8	Exercises: 5 min	Exercises: Trapezius stretches (3 × 30 s)/*SCOM stretches* (3 × 30 s) Neck extensor stretches (3 × 30 s)/*Neck flexion RCS:* 5 × (3–5 s) TM: Mobilization or HVLA	2/weeks (TM) Exercises: daily *(2 weeks)*	/	Home exercises	/	/
						Home exercises + TM		
Barassi et al. [7]	6	30 min	Muscle pressures: Maintaining pressure of approximately 3 kg and gradually releasing without abruptly stopping contact 5 patients: SCOM + *levator scapulae* 2 patients: *quadratus lumborum* + plantar region of the foot 3 patients: *trapezius, SCOM, levator scapulae, quadratus lumborum*	1/week *(8 weeks)*	/	Lumbar massage	/	/
						Prolonged muscle pressures		
Bialosky et al. (2014) [12]	8	/	TM: DL with contralateral lumbar rotation then HVLA; 2 times/side Placebo: Neutral spine push without rotation and no HVLA applied Placebo +: “The manual therapy you will receive has shown a significant reduction in low back pain in many people” + placebo Control: Sitting for 5 min, waiting	3/week *(2 weeks)*	/	Manual therapy	/	/
						Placebo		
						Placebo +		
						Control		
Fisher et al. (2016) [9]	7	Mob: 30 s	Mob: Hold ankle in traction for 30 s TM: Caudal directed ankle HVLA	One time session	/	Mobilization	/	/
						Manual therapy (HVLA)		
Gay et al. (2014) [10]	8	Mob: 5 min TT: 5 min	TM: HVLA type (Grade V), Mob: 2 min at 1 Hz 1 min rest, then 2 min at 1 Hz on the lumbar region TT: light pressure on sacrum for 5 min	One time session	/	Manual therapy (HVLA)	/	/
						Mobilization (Grade III)		
						Therapeutic touch		
Haider et al. [13]	5	/	TM: 1 non-thrust mobilization + 3 different thrust techniques on thoracic region + exercises (Maitland type) For both groups: exercises Hot/cold Mobility exercises (flexion and extension with arms in front of the wall; shoulder flexion 90°; exercises with shoulder circles) Strengthening exercises: resistance with elbow flexed at 90°; shoulder elevation with weights between 1–4 kg Deeps with elbow extension	3/week *(2 weeks)*	/	Manual therapy (Maitland) + exercises	/	/
						Exercises		
La Touche et al. (2013) [14]	8	3 × (2 min TM/30 s rest) = 7 min	TM: Patient in supine position, cervical region in neutral. (C0–C3 region); application of posterior force on the patient’s forehead by the therapist with the anterior part of their shoulder, both hands supporting the cervical region. Mobilization at 0.5 Hz Placebo: Same position but no force applied, simple contact maintained	3/week *(2 weeks)*	/	Manual therapy	/	ANS measurement immediately after the technique; 5 minutes later: VAS + PPT
						Placebo		
Peña-Salinas et al. (2017) [15]	8	/	TM: Patient lying down, therapist behind. Therapist applies force with their metacarpophalangeal joint of the right index finger towards the 1st rib; right thumb in the supraspinous fossa. The other hand supports the patient’s head with ipsilateral tilt + contralateral rotation. Inferomedial pressure applied to the 1st rib. Placebo: Same position without applying force	One time session	/	Manual therapy (HVLA)	/	/
						Placebo		
Rodrigues et al. (2021) [16]	7	3 min	TM: Patient lying on their back, arms across the chest. HVLA on the anteroposterior thoracic region. If no audible sound: maneuver repeated (max: 2/patients) Mob: Patient lying on their back, therapist’s pressure on the sternum, other hand supporting the patient’s neck. Longitudinal pressure directed caudally on the sternum. Once tissue barrier felt: 2 min at 0.3–0.4 Hz. Placebo: 2 min of ultrasound without current on the thoracic region	One time session	/	Manual therapy (HVLA)	/	/
						Mobilization		
						Placebo		
Sillevis et al. (2011) [17]	5	/	TM: HVLA T3–T4, anterior-posterior manipulation on the back, arms across the chest. Mob: Same position, but instructed to associate breathing; therapist’s 3-s compression during expiration	One time session	/	Manual therapy (HVLA)	/	Diameter measurement after intervention + measurement 4 min later
						Mobilization		
Weber et al. (2019) [18]	8	<5 min	TM: HVLA anterior-posterior T4–T5, patient lying on their back, arms crossed on the chest. Placebo: Same position but minimal pressure	One time session	/	Manual therapy (HVLA)	/	/
						Placebo		

Daily = every day; TM = manual therapy; HVLA = high veolocity, low amplitude; SCOM = sternocleidomastoid; DL = side-lying; Mob = mobilization; TT = therapeutic touch; / = no data available; TM = manual therapy; supine position = lying in the back; therapist = physiotherapist; ANS = autonomic nervous system; VAS = pain evaluation scale; PPT = pressure pain threshold; HVLA = high velocity, low amplitude.

**Table A4:** Characteristics of the study population included.

Studies	Pedro Scale	Population «Baseline»		**Samples**	
		Inclusion Criteria	Population Data		
Bakken et al. [6]	8	>18 years with recurrent (at least 1 previous episode) or persistent (>6 months) neck pain No chiropractic treatment in the last 3 months	Average age 57; 55% women; start pain (4/10); +80% neck pain > many years; 80% low risk of chronicity (Starback); 65% working people	Group: MT (HVLA or mobilization) + home exercises	N = 62
				Group: home exercises	N = 61
Barassi et al. [7]	6	Spinal pain between the ages of 20–29	Average age 25	Group: MT (prolonged muscular pressure)	N = 10
				Group: lumbar massage	N = 10
Bialosky et al. (2014) [12]	8	Between 18 and 60 years of age with low back pain > 4/10 in the last 24 h.	70% women, Average age 31, SMT: 12 weeks of pain Placebo: 24 weeks of pain Placebo +: 36 weeks of pain Control: 4 weeks of pain	Group: MT	N = 28
				Group: MT placebo	N = 27
				Group: MT placebo + positive message	N = 27
				Group: no interventions	N = 28
Fisher et al. (2016) [9]	7	Between 18 and 60 years of age with a history of ankle sprains (>2 weeks), and an ankle functional score below 24/48.	Average score: 18.7 ± 5.6	Group: TM type HVLA	N = 15
				Group: Ankle mobilization	N = 15
Gay et al. (2014) [10]	8	21 years old on average with no current experience of low back pain, but performing a protocol prior to the study inducing low back pain. Return 48 h later to have an intervention according to the group.	70% women	Group: MT (HVLA)	N = 6
				Group: MT (grade III)	N = 8
				Group: therapeutic touch	N = 10
Haider et al. [13]	5	Subacromial pain for 2–3 months, aged 25–60 years.	Average age 50, 55% women, 50% right side; 45% left side; 5% both side; pain > 3 months for 40% of participants	Group: MT Maitland + exercises	N = 20
				Group: exercises	N = 20
La Touche et al. (2013) [14]	8	Craniocervical pain of myofascial origin localized to the cervical and masticatory muscles. 1st diagnosis of myofascial pain; bilateral pain (masseter, temporal, upper trapezius, suboccipital muscles), pain > 3 months and > 30/100; neck or shoulder pain provoked by cervical	/	Group: antero posterior cervical MT	N = 11
				Group: placebo	N = 11
Peña-Salinas et al. (2017) [15]	8	Cervical or cervico-brachial pain following MVA within 3 months. Positive lateral rotation flexion test	Average age 34, 55% women	Group: TM (1st rib)	N = 27
				Group: placebo	N = 26
Rodrigues et al. (2021) [16]	7	Musculoskeletal pain > 18 years old	Average age 45, 55% women, >50% make exercises; chronic pain for >80%; 15% chest pain; 45% lumbar pain Significant difference between groups for blood pressure.	Group: TM spinal HVLA	N = 19
				Group: TM myofascial	N = 20
				Group: ultrasound placebo	N = 20
Sillevis et al. (2011) [17]	5	Patients with chronic neck pain below T4, provoked by cervical movements. Age range 18–65.	+70% women, average age 44	Group: TM type HVLA	N = 50
				Group: mobilization	N = 50
Weber et al. (2019) [18]	8	Participants with acute or sub-acute neck pain (<6 weeks).	Average age 37, 65% women	Group: TM type HVLA	N = 12
				Group: placebo	N = 12

MT = manual therapy/HVLA = high velocity low amplitude; grade III = mobilization grade without trust; Starback = questionnaire on the risk of chronic pain; / = no data.
